# Ultrastructural Adaptation of the Cardiomyocyte to Chronic Mitral Regurgitation

**DOI:** 10.3389/fcvm.2021.714774

**Published:** 2021-10-18

**Authors:** Daniella Corporan, Ana Segura, Muralidhar Padala

**Affiliations:** ^1^Structural Heart Research and Innovation Laboratory, Carlyle Fraser Heart Center, Emory University Hospital Midtown, Atlanta, GE, United States; ^2^Division of Cardiothoracic Surgery, Department of Surgery, School of Medicine, Emory University, Atlanta, GE, United States; ^3^Department of Pathology, Texas Heart Institute, Houston, TX, United States

**Keywords:** primary mitral regurgitation, mitral valve prolapse, cardiac remodeling, mechanobiology, cytoskeleton

## Abstract

**Introduction:** Mitral regurgitation (MR) imposes volume overload on the left ventricle (LV) and elevates wall stress, triggering its adverse remodeling. Pronounced LV dilation, minimal wall thinning, and a gradual decline in cardiac ejection fraction (EF) are observed. The structural changes in the myocardium that define these gross, organ level remodeling are not known. Cardiomyocyte elongation and slippage have both been hypothesized, but neither are confirmed, nor are the changes to the cardiomyocyte structure known. Using a rodent model of MR, we used immunohistochemistry and transmission electron microscopy (TEM) to describe the ultrastructural remodeling of the cardiomyocyte.

**Methods:** Twenty-four male Sprague-Dawley rats (350–400 g) were assigned to two groups: group (1) rats induced with severe MR (*n* = 18) and group (2) control rats that were healthy and age and weight matched (*n* = 6). MR was induced in the beating heart using a 23-G ultrasound-guided, transapical needle to perforate the anterior mitral leaflet, and the rats were followed to 2, 10, and 20 weeks (*n* = 6/time-point). Echocardiography was performed to quantify MR severity and to measure LV volume and function at each time-point. Explanted myocardial tissue were examined with TEM and immunohistochemistry to investigate the ultrastructural changes.

**Results:** MR induced rapid and significant increase in end-diastolic volume (EDV), with a 50% increase by 2 weeks, compared with control. Rise in end-systolic volume (ESV) was more gradual; however, by 20 weeks, both EDV and ESV in MR rats were increased by 126% compared with control. A significant decline in EF was measured at 10 weeks of MR. At the ultrastructural level, as early as 2 weeks after MR, cardiomyocyte elongation and increase in cross-sectional area were observed. TEM depicted sarcomere shortening, with loss of Z-line and I-band. Desmin, a cytoskeletal protein that is uniformly distributed along the length of the cardiomyocyte, was disorganized and localized to the intercalated disc, in the rats induced with MR and not in the controls. In the rats with MR, the linear registry of the mitochondrial arrangement along the sarcomeres was lost, with mitochondrial fragmentation, aggregation around the nucleus, and irregularities in the cristae.

**Discussion:** In the setting of chronic mitral regurgitation, LV dilatation occured by cardiomyocyte elongation, which manifests at the subcellular level as distinct ultrastructural alterations of the sarcomere, cytoskeleton, and mitochondria. Since the cytoskeleton not only provides tensegrity but has functional consequences on myocyte function, further investigation into the impact of cytoskeletal remodeling on progressive heart failure or recovery of function upon correcting the valve lesion are needed.

## Introduction

Mitral regurgitation (MR) is the most frequently diagnosed heart valve lesion in the USA, which occurs largely due to mitral valve prolapse (1, 2). Loss of mitral valve competence causes backflow of blood from the left ventricle (LV) into the left atrium (LA) in each heartbeat, which combined with the constant venous return regulated by the peripheral circulation, increases the total volume of blood filling the heart. The elevated blood volume returning to the LV imposes a chronic low-pressure, volume overload that increases chamber wall stresses, providing a trigger for the myocardium to adapt and adversely remodel (3, 4).

Ventricular remodeling secondary to MR has been studied at the organ level in preclinical rodent and dog models, and in humans (5–9). At the organ level, rapid and profound chamber dilation occurs upon the onset of MR, with a gradual decline in cardiac function, in animal models and in patients. In patients, symptomatic heart failure occurs nearly 5–10 years after the onset of MR, if it is not reversed (10). In a rodent model of severe MR (11), we recently reported similar findings as in human patients, with rapid and significant ventricular dilatation upon MR onset, preservation of ejection fraction for up to 14 weeks, followed by a gradual but persistent decline in ejection fraction (9). However, end-systolic volume and load-independent parameters of contractility were significantly worsened prior to the decline in ejection fraction, indicating the ineffectiveness in capturing LV dysfunction using traditional cardiac function indices (9).

For such extensive geometric alterations to the LV to occur, they need to be paralleled by ultrastructural alterations in the myocardium. Ventricular dilatation can occur either from longitudinal elongation of the cardiomyocytes that contributes to overall increase in chamber circumference, or slippage of the cardiomyocytes that causes a rise in circumference due to changes in the cellular spatial arrangement, or both. In response to MR, canine cardiomyocytes which have an average sarcomere length of 2.07 μm are stretched (12). However, their increase in length occured while preserving individual sarcomeric unit lengths by serial addition of sarcomeres at the intercalated discs (13–15). Slippage of cardiomyocytes is a prevalent hypothesis, but the arrangement of myocytes in the tissue makes slippage an unlikely process. Linzbach et al. reported in hearts from multiple species that the myocytes are arranged in a branching pattern (16), which makes lateral slippage of cardiomyocyte counterintuitive. There is no experimental evidence to date that myocyte slippage governs ventricular dilatation in MR.

Thus, to discern the ultrastructural changes that occur in the cardiomyocytes in the setting of chronic MR, in this study, we used a controlled model of MR in the rodent, and used a combination of transmission electron microscopy and immunohistochemistry to map cellular and subcellular changes.

## Materials and Methods

### Ethical Statement and Study Design

The study protocol was approved by the Institutional Animal Care and Use Committee at Emory University, and all surgical procedures were performed in accordance with the NIH guidelines for use of animals in research. Male rats were used in this study as our previous work on LV remodeling was in male rats, which was used for comparison (9). Thus, this dataset is relevant to male rats only and does not consider for differences in biological sex. Adult, male, Sprague-Dawley rats (*n* = 24, 350–400 g) were purchased from Envigo (Indianapolis, IN, USA) and housed in cages in a temperature- and humidity-controlled environment with 12:12 h light-dark cycle at an Association for Assessment and Accreditation of Laboratory Animal Care (AAALAC)-accredited facility. Throughout the in-life period of the study, rats had continuous access to standard rat chow and drinking water. Rats were assigned to two groups—(group 1) mitral regurgitation (*n* = 18) and (group 2) control rats that were healthy and age and weight matched (*n* = 6). In group 1, six rats each were terminated at 2, 10, and 20 weeks after the onset of the valve lesion, for assessment of the myocardial ultrastructure.

### Surgical Model of Mitral Regurgitation

The procedure to induce MR with ultrasound guidance was previously described (9, 11). In this model, the procedural survival is 92% (9). The surgery was performed with the rats sedated, intubated, and mechanically ventilated with 2.5% isoflurane in 100% oxygen. Upon placing the rat in right decubitus position, a left thoracotomy was performed in the fourth intercostal space to expose the apex of the heart. A pericardiotomy was performed and a purse string was placed on the apex, with a 6–0 prolene suture (8307H, Ethicon, Raritan, NJ, USA). An 8-Fr transesophageal echocardiographic (TEE) probe (8 MHz, Acunav, Biosense Webster, Diamond Bar, CA, USA) was advanced into the esophagus to obtain a two-chamber view of the heart. A 23-G needle with a proximal stopcock was flushed with saline and inserted through the apical purse string into the LV chamber. With ultrasound guidance, the needle tip was advanced toward the mitral valve and the anterior leaflet was perforated. The needle was then retracted, and MR was confirmed on color Doppler imaging immediately, and its severity was quantified with both color and spectral Doppler imaging. The surgery was completed by tightening the purse string suture, drying the chest with gauze, closing the thoracotomy in layers with 4–0 vicryl suture (J496H, Ethicon), and placing a temporary chest tube (SR-OX1651CA, IV catheter, Terumo, Biñan, Philippines) to evacuate effusions. Rats were recovered from surgery, and Burprenex (0.02 mg/kg, SQ) was administeted within 3 h of surgery. Carprofen (5 mg/kg, SQ) and gentamicin (6 mg/kg, SQ) were administered daily for 3 days after surgery.

### Echocardiographic Assessment of Mitral Regurgitation and Cardiac Function

Transesophageal echocardiography was performed at baseline and 2 weeks after surgery, to quantify MR severity in the rats in group 1. A high esophageal, two-chamber view of the left atrium and left ventricle were obtained to assess mitral flow and color Doppler for quantification of MR. MR was quantified using three approaches: (1) MR jet area (%) by tracing the regurgitant jet area on color Doppler and normalized to the left atrial area; (2) MR volume (μl) by measuring the MR jet velocity time integral on continuous wave Doppler and multiplying it by the area of the regurgitant orifice created from the 23-G needle (0.64 mm OD); (3) pulmonary flow reversal measured by taking the ratio of the pulmonary systolic and diastolic wave velocities. Transthoracic echocardiography (TTE) was performed at termination with a 21-MHz probe on the Visualsonics 2100 ultrasound system (Fujifilm Visualsonics Inc., Tokyo, Japan). Parasternal long-axis views were acquired to measure left ventricular volumes. The endocardial border of the LV was traced in diastole and systole on B-mode TTE images, and end-diastolic volume (EDV), end-systolic volume (ESV), and ejection fraction (EF) were calculated. The long-axis and short-axis dimensions of the LV were measured, and sphericity was defined as the ratio of the two dimensions.

### Histology of the Myocardium

Upon termination, the hearts from the rats with MR that were terminated at 2 and 10 weeks, and the age- and weight-matched sham rats (*n* = 3/group) were perfusion fixed with 10% formalin. A 16-G needle was inserted into the aorta, with its tip placed above the aortic valve, and formalin was injected with a syringe to perfuse the myocardium through the coronary arteries. The hearts were left in 10% formalin for 24 h, and then transferred to 70% ethanol. Histopathology was performed at an independent site, Alizee Pathology (Thurmont, Maryland), where tissue sectioning, processing, paraffin embedding, hematoxylin, and eosin staining and Gomori's elastin trichrome staining were performed. Slides were imaged with the NanoZoomer digital slide scanner (Hamamatsu, C13210-01) at the Winship Pathology Core at Emory University. From each cross-section of the hearts in each group, the cardiomyocyte cross-sectional area was measured using ImageJ software (NIH, USA). Individual cells were identified, and their border was traced and the pixels within the border were calibrated against a scale to measure the area. In each image, cardiomyocyte cross-sectional area was measured from 20 or more different cells, and the data were averaged.

### Immunohistochemistry to Investigate the Cytoskeleton

Paraffin-embedded blocks of the hearts were sectioned, and LV tissues were stained from all the groups. Four-micrometer-thick midventricular cross sections were prepared using a microtome and transferred to glass slides (Fisher, 22-037-246). Tissues were deparaffinized in two changes of Xylene for 10 min each, then rehydrated with one change of 100% ethanol for 5 min, another change of 70% ethanol for 5 min, and lastly ddH_2_0 for 5 min or until staining. Antigen retrieval was performed using citrate buffer (Sigma, C9999) at a pH of 6; 1× citrate buffer in ddH_2_0 was heated to 100°C in an oven and then slides were immersed into the buffer in a staining dish for 20 min and then cooled to room temperature for 40 min. Slides were then marked with a hydrophobic pen (Sigma, Z377821) and permeabilized with 0.1% Triton X-100 in 1× PBS (Thermo, 10010049) for 5 min at room temperature. Slides were blocked with 10% donkey serum (Vector Laboratories, 017-000-121), 10% goat serum (Vector Laboratories, S-1000-20), and 1% bovine serum albumin (Sigma, A2153-100G) in 1× PBS for 2 h at room temperature. Primary antibody solutions were prepared using diluted blocking buffer (1:10) and a mouse monoclonal antidesmin (Sigma Cat# MABT535, RRID: AB_2891228) (1:250). Primary antibody was added to the slides and incubated overnight at 4°C. Slides were rinsed thrice in 1× PBS for 5 min each. Secondary antibody solutions were prepared using diluted blocking buffer (1:10) with donkey antimouse IgG H&L Alexa Fluor® 647 (Abcam Cat# ab150115, RRID:AB_2687948) (1:250). Secondary antibody was added to the slides and incubated for 1 h at room temperature in a dark room to avoid photobleaching, followed by three washes in 1× PBS for 5 min each. Slides were then counterstained with DAPI (1:100) in 1× PBS for 15 min, followed by three washes in 1× PBS for 5 min each, and finally cover-slipped using the FluorSave reagent (Sigma, 345789-20ML). Slides were stored in the dark until imaging, which was performed on an LSM 710 NLO confocal microscope, with a Zeiss 710 confocal scan head mounted on an Axio Observer Z1 inverted microscope stage. Cardiomyocyte length was measured using ImageJ software (NIH, USA).

### Transmission Electron Microscopy

Whole hearts from the MR 2- and 20-week groups and sham (*n* = 3/group) were extracted, rinsed, and perfused with 1× PBS to flush the blood out of the coronary arteries. Hearts were then perfused through the coronary arteries and fixed with 3% glutaraldehyde in 1× PBS and stored in the same solution at 4°C until transmission electron microscopy was performed at the cardiovascular pathology core lab at Texas Heart Institute (Houston, TX, USA). Small sections of the LV wall were washed in 1 M phosphate buffer (pH 7.3), postfixed in 1% osmium tetroxide for 1 h and dehydrated through a series of graded alcohol baths. Tissue samples were infiltrated with acetone and Epon 812 plastic resin and embedded in plastic molds with 100% Epon 812 plastic resin. Thick sections (1 μm) were cut from blocks and placed on glass slides using a Leica EM UC7 ultra microtome. Sections were stained with Toluidine Blue. Ultra-thin sections (70–80 nm) were cut from blocks using Leica EM UC7 ultra microtome. Sections were mounted on 100 mesh copper grids. Grids were stained with 2% uranyl acetate and Reynold's lead stain. Grids were imaged on a JEOL JEM-1230 electron microscope. Images were captured with AMT XR80 digital camera at ×300, ×500, ×1,000, and ×2,000. Sarcomere lengths were measured using ImageJ software (NIH, USA).

### Statistical Analysis

Statistical analysis of the quantitative data was performed in Prism GraphPad v7.0a (LaJolla, CA, USA). Data were tested for normality using the Shapiro-Wilk normality test. Data which passed the normality test were compared with one-way ANOVA followed by Tukey's *post-hoc* test for multiple comparisons. Data which failed the normality test were compared using a non-parametric, Kruskal-Wallis test with Dunn's multiple comparison test.

## Results

### Mitral Regurgitation Severity and Left Ventricular Dysfunction

MR severity at 2 weeks after surgery is tabulated in Table 1. Average MR jet area normalized to left atrial area was 37.43 ± 7.55%, and average MR volume was 125.00 ± 20.55 μl in all the MR groups. After inducing MR, the pulmonary venous flow systolic to diastolic wave ratio decreased from 0.84 in the control group, and ranged from −1.01 to −1.99 in the MR groups. Left ventricular end-diastolic volume increased after the onset of severe MR at each time-point. After only 2 weeks of MR, end-diastolic volume was increased by 50% compared with controls. By 10 weeks, end-diastolic volume was increased by 62% compared with controls (*p* = 0.0021). After 20 weeks of MR, end-diastolic volume was increased by 126% compared with controls. End-systolic volume increased by nearly 28% after 2 weeks of MR. By 10 weeks, end-systolic volume was increased by 76% compared with controls (*p* = 0.016) and by 20 weeks, end-systolic volume was increased by 126% compared with controls (*p* = 0.0089). Stroke volumes were also elevated after the onset of MR. Ejection fraction slightly increased from 64.93 to 70.08% after 2 weeks of MR, owing to the acute reduction in afterload from MR. By 10 weeks, ejection fraction declined to 61.69%, which was significantly lower compared with the 2-week data (*p* = 0.012).

**Table 1:** Echocardiographic parameters of MR severity and left ventricular chamber volumes in each experimental group.

	**Sham**	**MR 2 wk**	**MR 10 wk**	**MR 20 wk**
MR fraction (%)	0	38.71 ± 4.08^*^	35.39 ± 3.44	36.89 ± 2.36
MR volume (μL)	0	127.70 ± 7.56^*^	123.60 ± 11.92	121.10 ± 18.18
Pulmonary S/D ratio	0.84 ± 0.074	−1.35 ± 0.24	−1.01 ± 0.14^*^	−1.99 ± 0.47
End diastolic volume (μL)	421.10 ± 6.65	631.70 ± 39.34	681.70 ± 26.03^*^	952.60 ± 192.80
End systolic volume (μL)	147.70 ± 7.45	188.80 ± 12.31	260.20 ± 4.36^*^	333.80 ± 76.89^*^
Stroke volume (μL)	273.40 ± 4.74	442.90 ± 28.48^*^	421.50 ± 28.92	618.90 ± 120.8^*^
Ejection fraction (%)	64.93 ± 0.45	70.08 ± 0.75	61.69 ± 1.98^#^	65.19 ± 2.86
Sphericity index (%)	51.71 ± 2.04	58.65 ± 1.75	54.72 ± 1.26	63.46 ± 3.71^*^

*Data are represented as mean ± standard error. P-values p < 0.05 were considered to be statistically significant*.

* *p < 0.05 vs. sham;*

# *p < 0.05 vs. MR 2 wk*.

### Gross Morphology of the Left Ventricle

**Figure 1:**
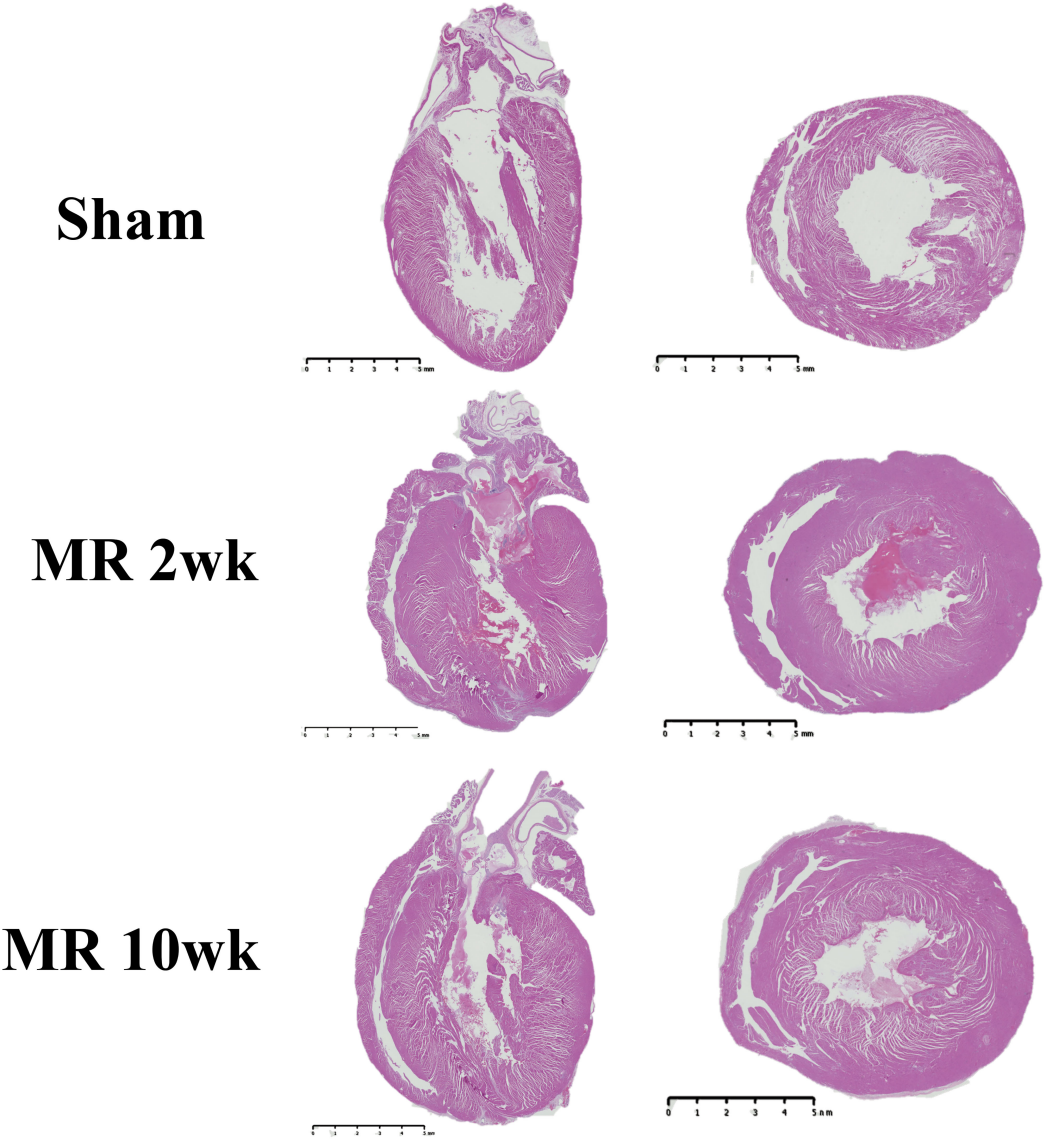
Representative images of the whole heart and mid-left ventricular cross-sectional morphology from H&E staining in the sham, MR 2-week, and MR 10-week groups. Left, a two- or four-chamber longitudinal view of the heart in the experimental groups. Right, a cross-sectional view of the midventricular level. Wall thickness in the sham group appears to be less than the MR 2- and MR 10-week groups.

**Figure 2:**
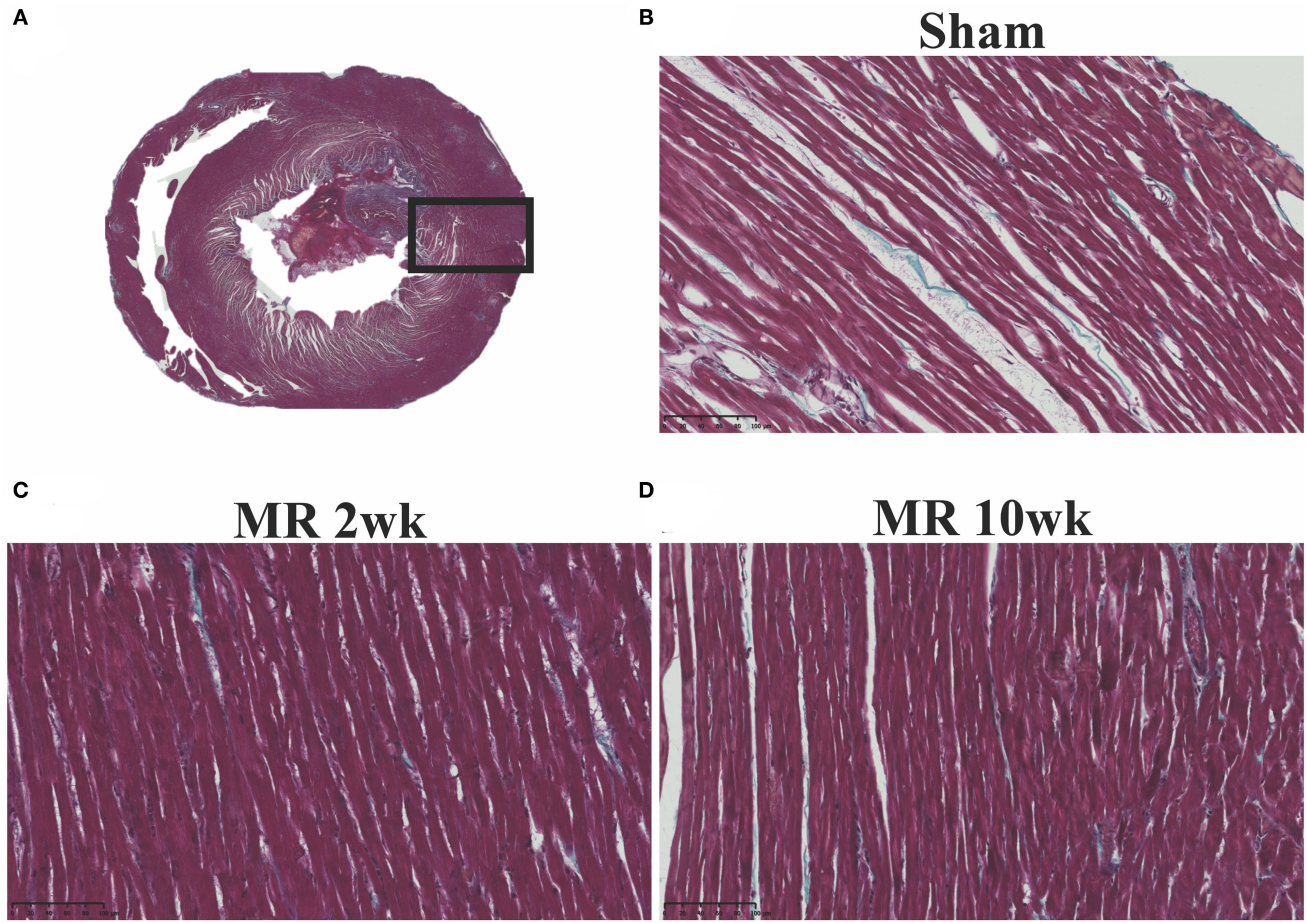
**(A)** Representative cross-sectional view of the midventricular level of the entire rat heart on gomori elastin trichrome staining. **(B)** Representative ×20 image of longitudinally aligned fibers from the from the sham group. **(C)** MR 2-week group; and **(D)** MR 10-week groups. Intersitial collagen between the fibers are shown in blue in each group, and the density of the fibers appears to be increased in the MR 2- and MR 10-week groups compared with sham.

**Figure 3:**
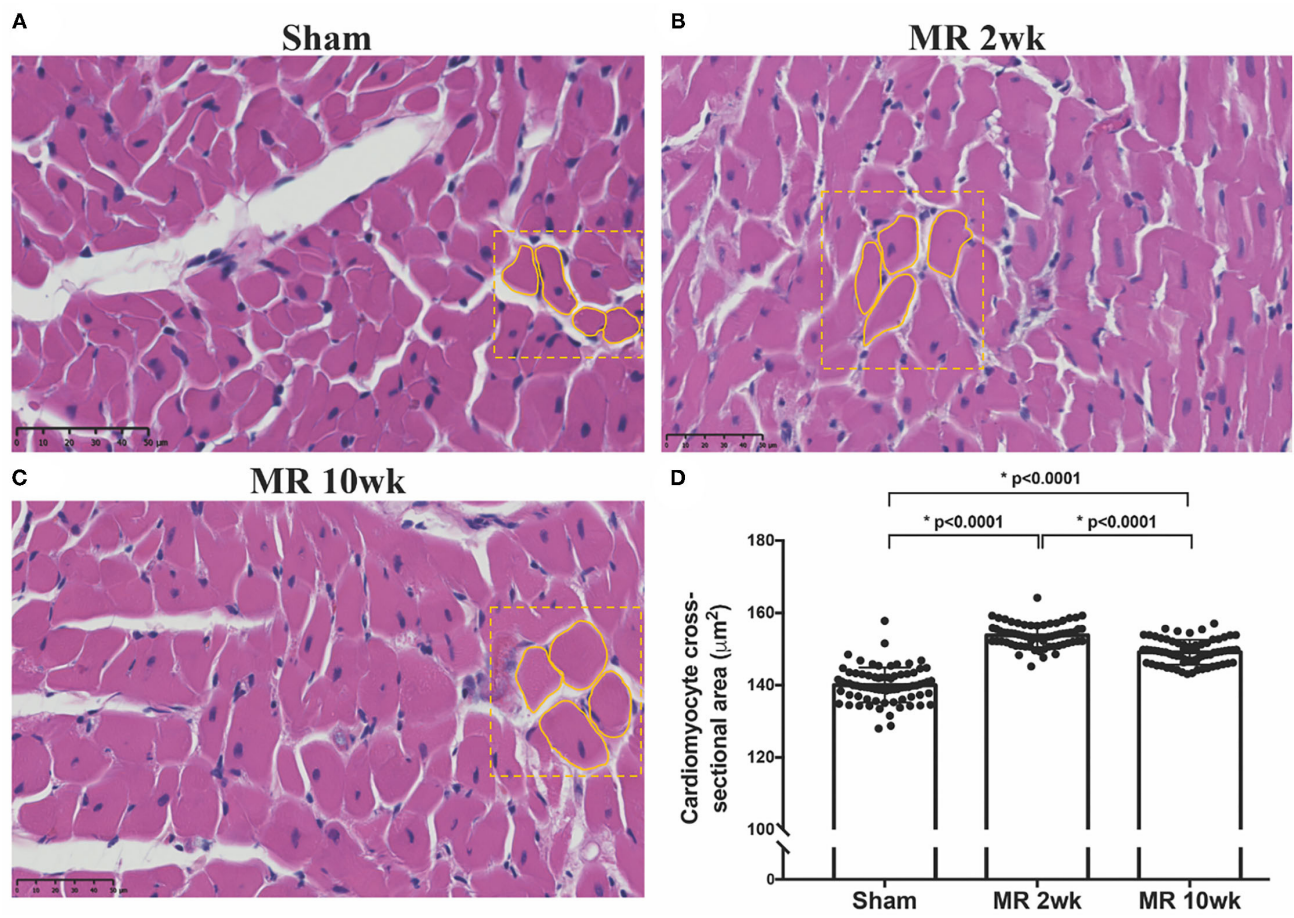
Cross-sectional view of the cardiomyocytes on H&E staining in the three samples from the **(A)** sham, **(B)** MR 2-week, and **(C)** MR 10-week groups. **(D)** Average cardiomyocyte cross-sectional area (CSA) quantified from the H&E staining in each group using ImageJ. Data are represented as mean ± 1 standard deviation, and *p*-values <0.05 were considered statistically significant. Both the MR 2- and 10-week group had a significantly higher cardiomyocyte CSA compared with sham (*p* < 0.0001).

The gross morphology of the heart and midventricular cross section in both groups are shown in Figure 1. In the hearts from the control group, the longitudinal and cross-sectional views of the LV wall are thinner, compared with the rats with MR. Thickness of the interventricular septum and the LV lateral wall were similar in the control group; however, in the hearts with MR, the interventricular septal wall was thinner compared with the lateral wall. Gomori elastin trichrome-stained images from the midventricular level are shown in Figure 2A. In the hearts from the control group, parallel alignment of myocardial fibers and some interstitial collagen is evident, with dispersed interstitial spaces throughout the tissue (Figure 2B). In the hearts with MR, the interstitial spaces are reduced, with the myocytes occupying a larger area of the muscle. In the small interstial spaces still present, the collagen fibers are denser (Figures 2C,D). Cardiomyocyte cross-sectional area (CSA) is shown in the H&E-stained LV tissue in Figures 3A–C. The average cardiomyocyte CSA in the control group was 140.00 ± 4.85 μm^2^ (Figure 3D). In the 2-week MR hearts, the average cardiomyocyte CSA was 153.80 ± 3.26 μm^2^, which was significantly higher compared with the control (*p* < 0.0001). In the 10-week MR hearts, the average cardiomyocyte CSA was 149.10 ± 3.42 μm^2^, which was higher compared with the control (*p* < 0.0001), however, was lower compared with the MR 2-week group (*p* < 0.0001).

### Cardiomyocyte Cytoskeletal Alterations With Mitral Regurgitation

**Figure 4:**
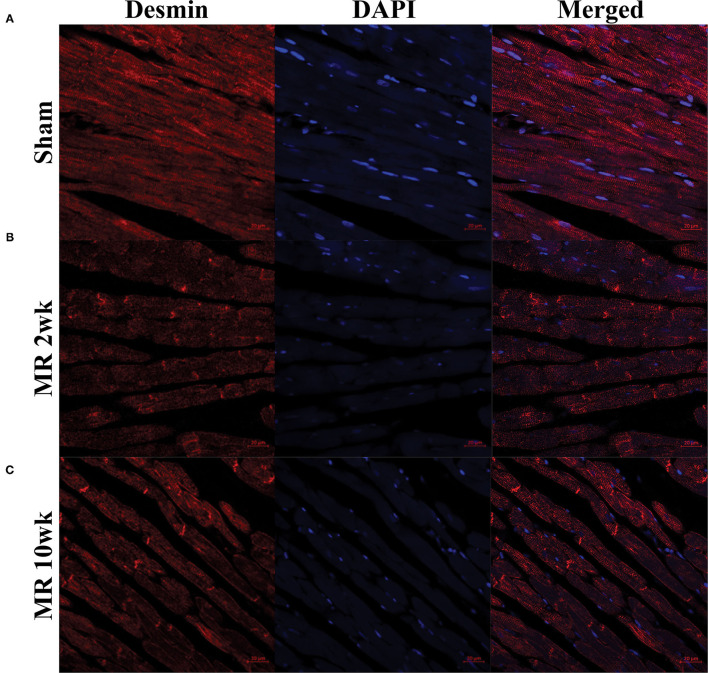
Immunohistochemistry of the intracellular cytoskeleton protein, desmin, in sham **(A)** and MR hearts **(B,C)** at ×63 magnification. Staining of desmin alone is shown in the left column for each group; DAPI staining is shown in the middle column; merged staining is shown in the right column. In the sham group, desmin is regularly distributed throughout the myocytes and concentrated at the Z-discs. In the MR 2- and 10-week group, desmin staining is irregular and focally concentrated at the intercalated disc region and not the Z-discs.

The distribution and organization of the cytoskeletal protein, desmin, along the length of the cardiomyocyte is shown in the immunohistochemistry images in Figure 4. In the control group, desmin is organized along the length of the cardiomyocytes, with condensed staining observed at the Z-disk (Figure 4A). After 2 weeks of MR, desmin staining was less organized along the length of the cardiomyocytes (Figure 4B). Irregular desmin staining was observed along the cells and at the Z-disks but was focally condensed at the intercalated disks. After 10 weeks of MR, desmin was largely concentrated at the intercalated disks; however, desmin was also more regularly organized at the Z-disks compared with 2 weeks but was still not comparable with controls (Figure 4C).

### Cardiomyocyte Ultrastructural Alterations After Mitral Regurgitation

**Figure 5:**
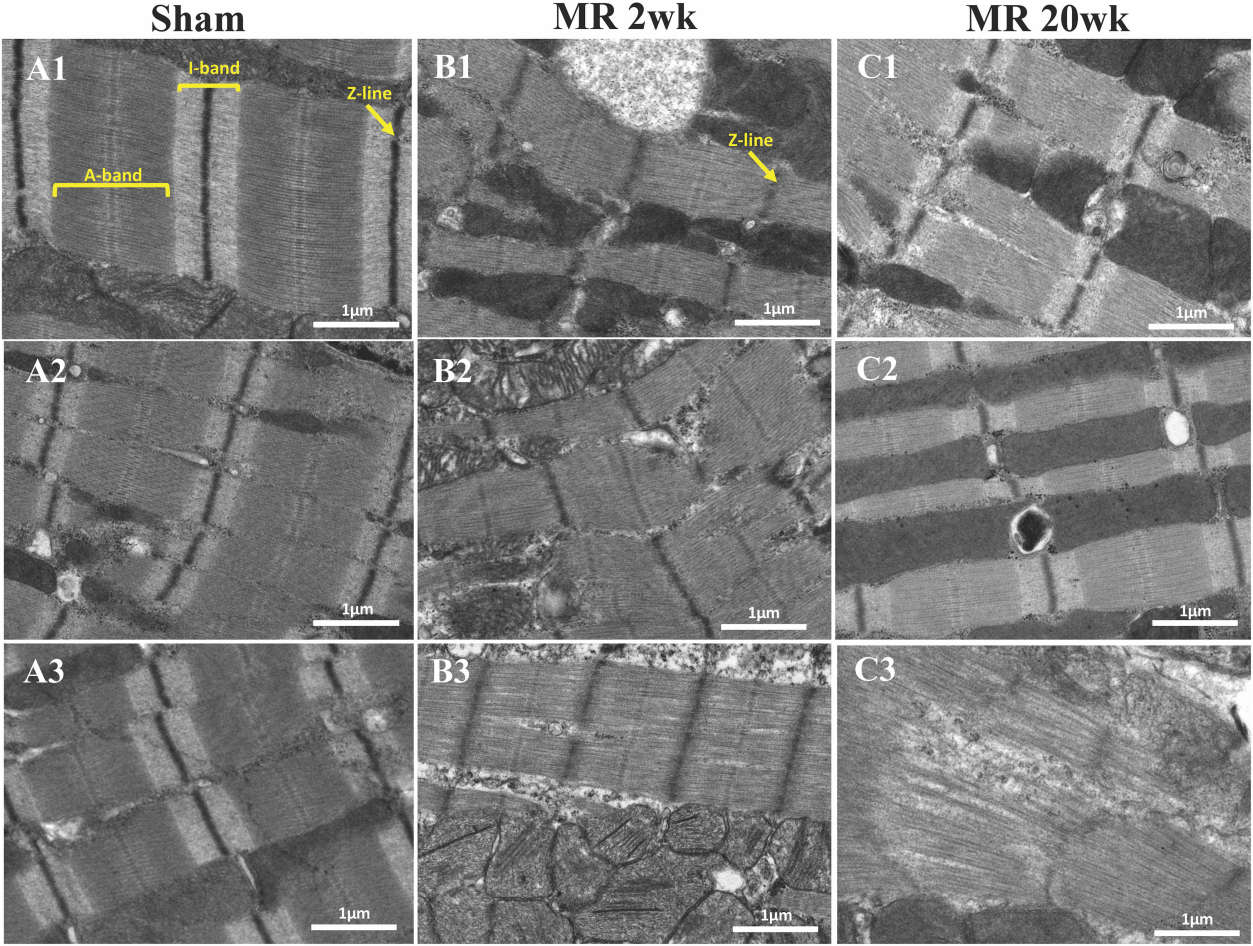
Transmission electron microscopy images detailing the sarcomere structure, intracellular organelles, and their spatial organization. **(A1–A3)** Sarcomere structure from three different rats, depicting an organized sarcomeric network, with clearly visible and condensed Z-line, I-band, and A-band. A linear registry of mitochondria that are interspersed parallel to the myofibrils and adjacent to the sarcomeres are evident. **(B1–B3)** After 2 weeks of mitral regurgitation, ultrastructural changes are clearly evident. The I-band was not evident, and the density of the Z-band was reduced. The mitochondria interspersed between the sarcomeres were fragmented and disorganized. **(C1–C3)** At 20 weeks of mitral regurgitation, damage to the sarcomeres was evident.

**Figure 6:**
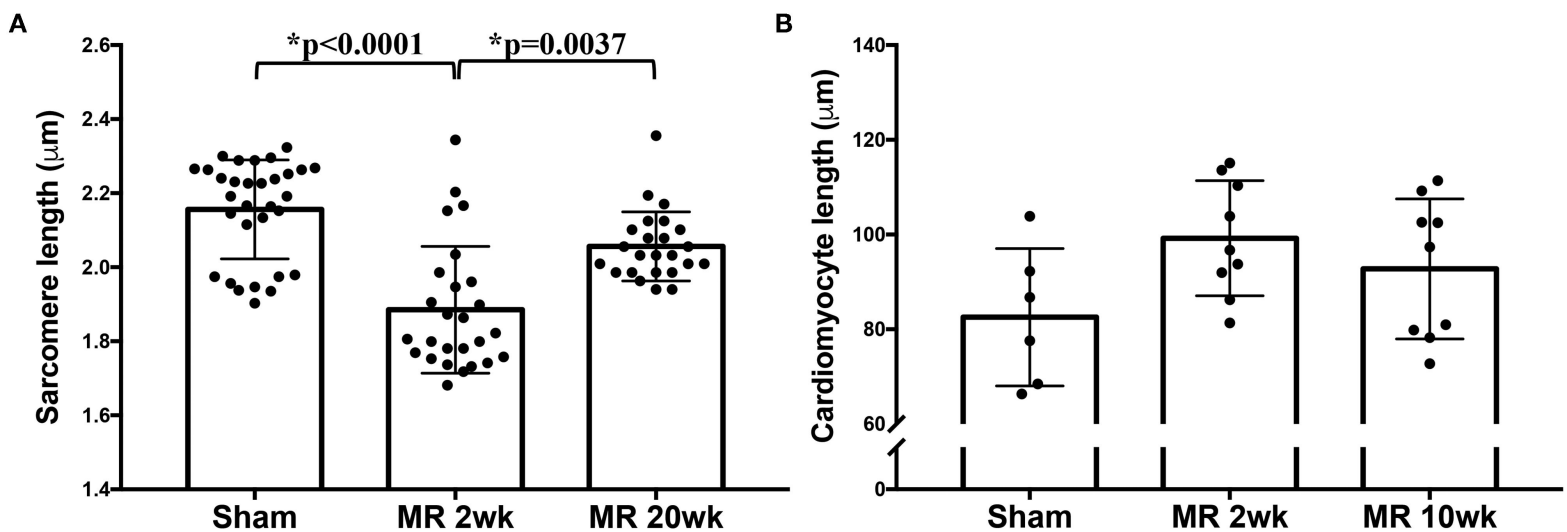
**(A)** Sarcomere length measured from TEM images of the sham, MR 2-week, and MR 20-week groups. **(B)** Cardiomyocyte length measured from immunohistochemistry images from the sham, MR 2-week, and MR 20-week groups. Data are represented as mean ± standard deviation. *p* < 0.05 represent statistical significance.

High-resolution TEM imaging of the cardiomyocyte sarcomeric structure in both groups is shown in Figure 5. In the control group, the sarcomeric structure was normal and consisted of typical features such as the dark A-band of thick filaments, light I-band of thin filaments, M-line, and Z-line (Figure 5A). After 2 weeks of MR, there was a loss of I-band and reduced Z-line density compared with controls (Figure 5B). The myofibrillar organization is preserved throughout the sarcomeres, with the thick and thin filaments spanning across Z-lines. Overall cardiomyocyte length increased after 2 weeks of MR compared with control (*p* = 0.078); however, the sarcomere length was significantly lower in the MR 2-week group compared with sham (*p* < 0.0001) (Figure 6). After 20 weeks of MR, the A-band, I-band, and Z-line structures were preserved in two of the three samples (Figures 5C1,C2). In one sample, there was substantial loss of the sarcomeric structure including loss of the Z-line density and I-band features (Figure 5C3). The sarcomere length was not different compared with control after 20 weeks of MR but was significantly higher compared with the sarcomere length at 2 weeks (*p* = 0.0037) (Figure 6).

**Figure 7:**
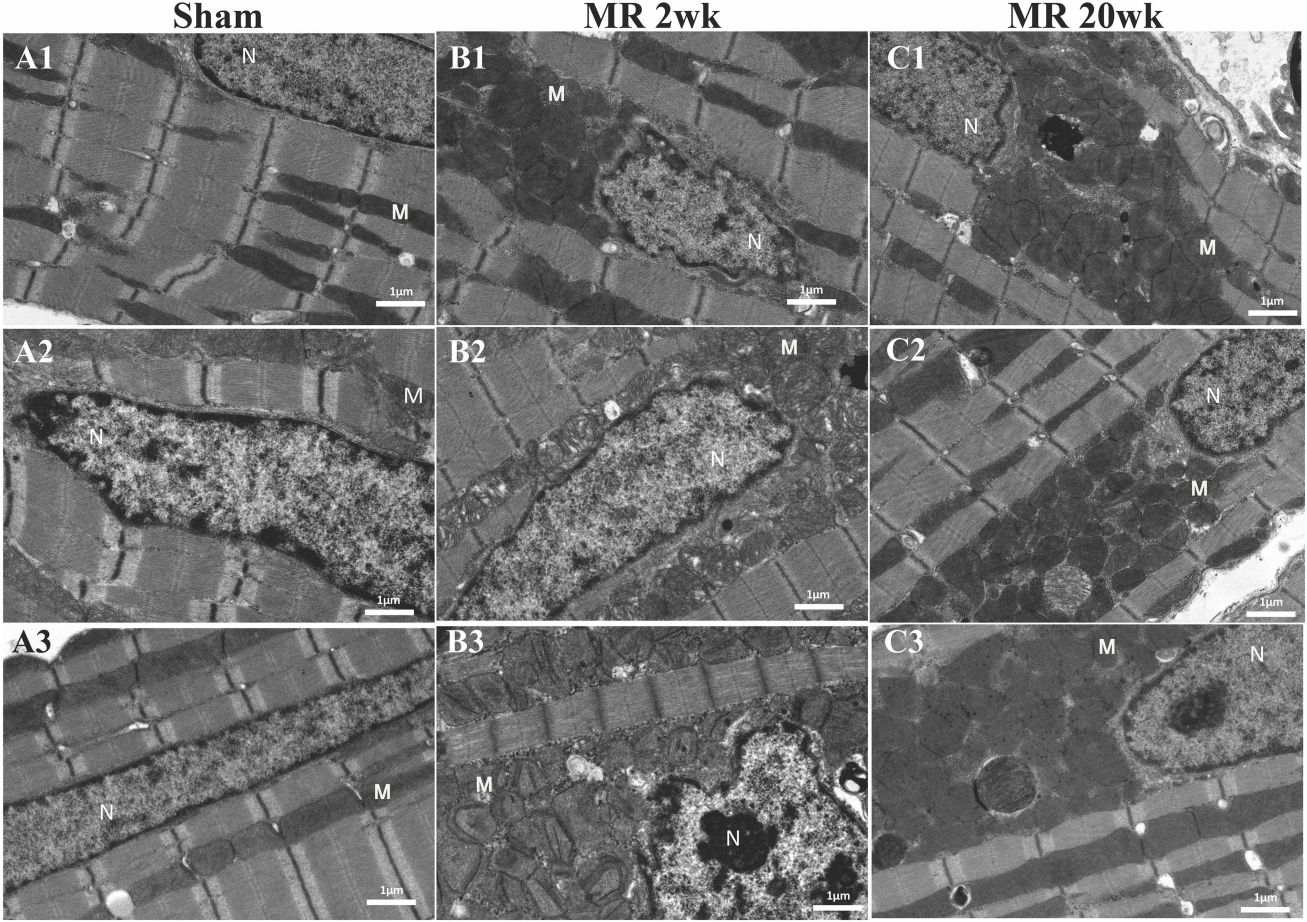
Transmission electron microscopy images detailing the mitochondrial organization in each rat left ventricle from the sham, MR 2-week, and MR 20-week groups. Representative images from three individual samples from different rats are shown in the sham group **(A1–A3)**, MR 2-week **(B1–B3)**, and MR 20-week group **(C1–C3)**. The nucleus (N) and mitochondria (M) is labeled in each image.

**Figure 8:**
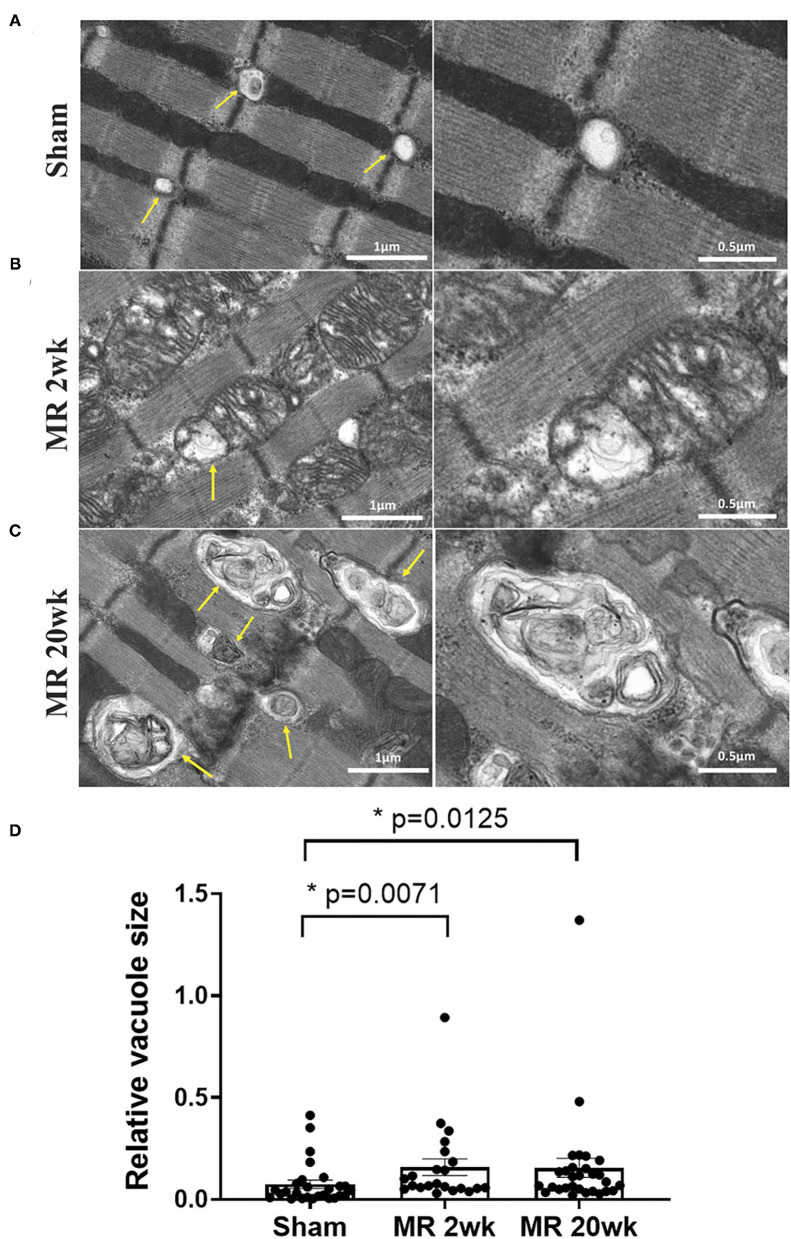
Transmission electron microscopy images detailing the presence and ultrastructure of vacuoles in each rat left ventricle from the sham **(A)**, MR 2-week **(B)**, and MR 20-week groups **(C)**. The vacuoles are depicted by yellow arrows in images. Sham rats had fewer vacuoles present within the myocardium, and when visualized, vacuoles were small in size and were located near the Z-disc. In the MR samples at 2 and 20 weeks, there was a higher presence of vacuoles of increased size which were filled with electron-dense material. Vacuoles were present near mitochondria, Z-discs, and the intercalated disc region. **(D)** Quantitative analysis of relative vacuole size measured using ImageJ. Data are represented as median with interquartile range (IQR: 25–75%). The non-parametric data were compared using the Kruskal-Wallis test. *p* < 0.05 was considered statistically significant.

**Figure 9:**
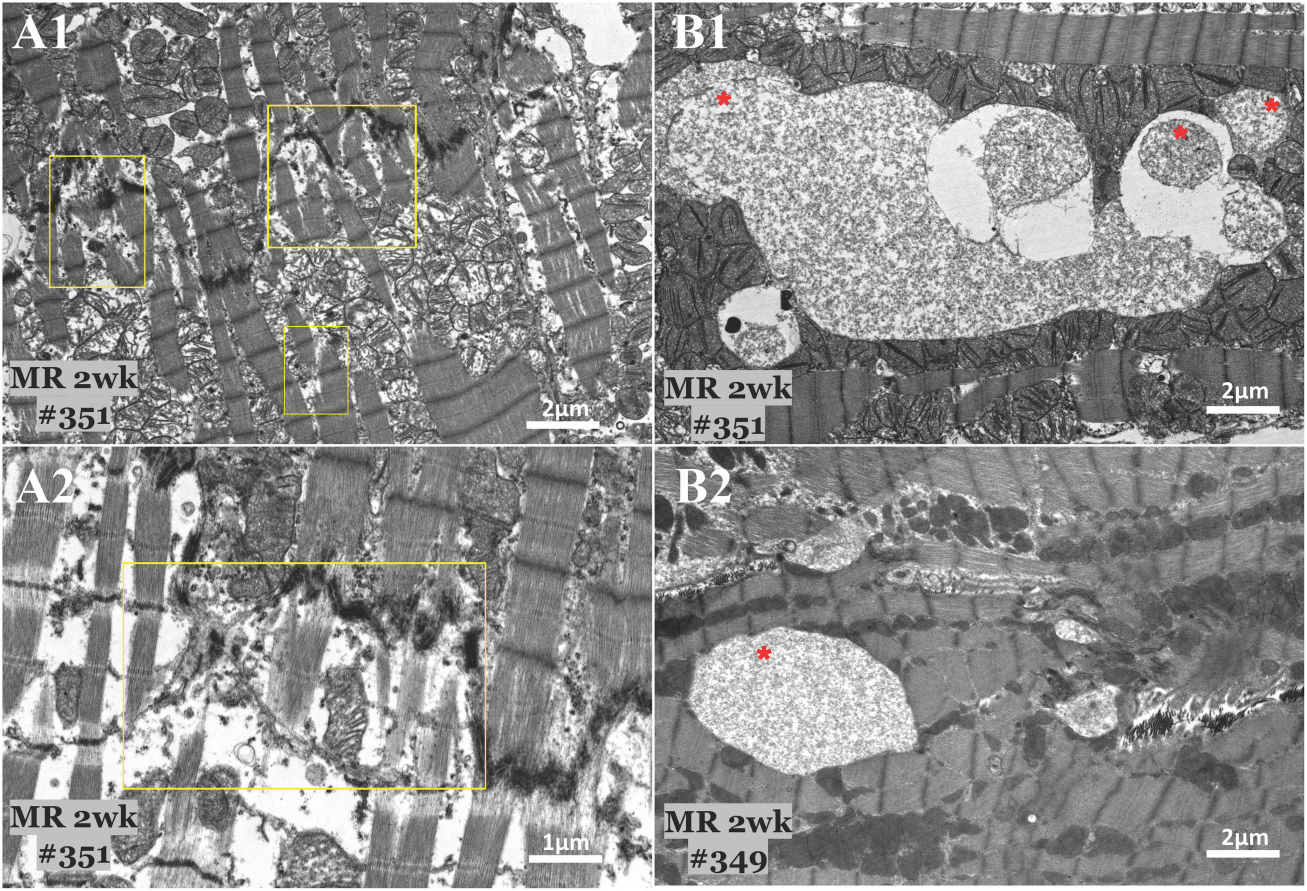
Transmission electron microscopy images detailing focal areas of myocytolysis and fragmentation **(A)** and glycogen accumulation **(B)** in two MR 2-week samples. Myocytoloysis and fragmentation of the sarcomeres were observed in some areas of the sample, which are depicted in the yellow rectangle. Some areas also showed significant accumulation of glycogen granules between the sarcomeres and within the mitochondria, which are depicted by the red asterisk.

The mitochondrial organization within the cardiomyocyte is shown in Figure 7. In the hearts from the control group, mitochondria were aligned as a linear registry along the sarcomeres, without clustering around intracellular organelles (Figure 7A). In the tissue from the 2-week MR group, there was a higher density of mitochondria surrounding and interspersing the sarcomeres (Figure 7B). At several areas within the cells, there was a loss of the linear registry of the mitochondria, with substantial clustering around the nucleus. In the MR 20-week tissue, the mitochondria were enlarged with irregular and disorganized cristae (Figure 7C). The mitochondria were also clustered and aggregated around the sarcomeres and the nucleus. Large vacuoles were observed in cardiomyocytes in the tissues from the animals with MR (Figure 8). In the control tissues, the presence of vacuoles was lower and the size of the vacuoles was considerably smaller than MR hearts. Quantification of relative vacuole size in the MR 2- and 20-week group was significantly higher compared with sham (0.072 (IQR: 0.054–0.19) vs. 0.037 (IQR: 0.01–0.078), *p* = 0.0071; 0.086 (IQR: 0.046–0.15) vs. 0.037 (IQR: 0.01–0.078), *p* = 0.0125) (Figure 8D). In the rat hearts with MR, vacuoles were observed near the Z-line, mitochondria, and intercalated disks. The vacuoles had double-membrane-bound bodies with electron-dense material or internalized debris. In the tissues from the MR 2-week group, focal areas of noticeable myocytolysis and fragmentation of the myofibrils were observed in one sample (Figure 9A) and substantial glycogen accumulation between the mitochondria (Figure 9B1) and the sarcomeres was noted (Figure 9B2).

## Discussion

In this study, we investigated the ultrastructural alterations in the myocardium in a rodent model of MR. After the onset of MR, LV dilation occurred within 2 weeks, though reduction in ejection fraction was more gradual, with a statistically significant reduction only after 8 weeks after the onset of MR. The diastolic ventricular volume increase was rapid and large, increasing by 50% within 2 weeks of MR. By 20 weeks, the rise in end-diastolic volume was 126%. End-systolic volume increase, indicative of contractile dysfunction, was only 28% after 2 weeks, but at 126% by 20 weeks. The ventricular remodeling observed in this model parallels that reported in other animal models (17–19) and in patients with MR (5, 20, 21). The mechanistic basis for ventricular dilatation is unclear, though both myocyte elongation and slippage have been hypothesized. Preservation of ejection fraction for prolonged periods in the setting of MR is expected, as mitral valve insufficiency into the left atrium, has an afterload reducing effect. Additionally, the low impedance from the left atrium, allows ventricular ejection despite end of electrical systole, contributing to an overall smaller end-systolic volume that elevates the ejection fraction. This phase of remodeling, typically termed the compensatory phase of remodeling, is observed in patients with MR as well (5, 8). Whether this phase is truly compensatory or if structural adaptations at the cellular level occur is not well-known.

Our data indicate that ventricular dilation after the onset of MR, was paralleled by elongation of the cardiomyocytes and an increase in cardiomyocyte cross-sectional area at both 2 and 10 weeks compared with the control group. Cardiomyocyte elongation after volume overload induced stretch was reported by Gerdes et al. (13), in an aortocaval fistula model (ACF) that is an exaggerated volume overload stimulus compared with MR. These findings were confirmed in other species as well in a similar ACF model (14, 22). Elongation of the cardiomyocyte as the mechanism contributing to ventricular dilatation is quite reasonable. Cardiomyocytes are aligned in series in each myofiber, and a collection of such myofibers form a longitudinal helical pattern in the ventricles. In the hearts with MR, the dilatation was spherical. The sphericity index increased over 20 weeks compared with sham (63.46 ± 3.71 vs. 51.7 ± 2.04%, *p* = 0.02), indicating an increase in both the short axis and long axis of the left ventricle, which can only occur if the cardiomyocytes have elongated, or have slipped without any elongation, thus adding gaps between the cells. This elongation of the cardiomyocytes has been hypothesized to accommodate the increased chamber volumes (23). Our data confirms that elongation is likely a significant contributor. However, at 2 weeks of MR, the cardiomyocytes only elongated by 20%, whereas the rise in end-diastolic volume was 50%. This discrepancy can likely be explained by the oblique arrangement of the myocytes to the long axis of the heart.

Surprisingly, our data demonstrate that cardiomyocyte elongation did not cause sarcomere stretching, despite the arrangement of the sarcomeres from one intercalated disk to the other, spanning the length of the cardiomyocyte. Measurement of sarcomeric lengths on electron microscopy demonstrated a decrease in the diastolic sarcomeric length in the hearts with MR. Such a decrease in sarcomere length, when myocyte elongation occurs, could be possibly attributed to the breakdown of structural arrangement within the sarcomere, including the thin filament region and supporting Z-disc. Magnified examination of the sarcomeric units indicated loss of thin filament I-band and fading of the Z-disk, indicating damage of this region of the sarcomere. The Z-disk connects the sarcomeres in series, whereas the I-band region of thin filaments within the sarcomeric unit, typically consists of the elastic PVEK segment of the thick titin protein, and governs its stretchability and thus the overall sarcomeric resting length as well (24). Recently, completely obliterating the mitral valve in mice demonstrated a similar finding with damage of the sarcomeres in these same regions (25). The proteins which interact with the Z-disc stabilize the sarcomeric structure of the myocyte, and help to maintain the distinct Z-line, M-line, H-band, A-band, and I-band features of the sarcomere. The loss of the I-band feature after 2 weeks of MR could be due to altered levels of titin or different titin isoforms, causing a disorganization of the Z-line and I-band and shortening of the I-band region in the passive relaxation state of the cardiomyocytes. The shorter N2B isoform of titin is known to increase the diastolic resting force of cardiomyocytes (26), thus a switch to the N2B isoform could explain the decreased sarcomere lengths observed after 2 weeks of MR. Although we did not specifically study titin or its isoforms in this study, previous work has shown titin isoform switching (27) and hypophosphorylation of titin (6) in the acute phase after volume overload. Thus, alterations in titin could potentially explain the decreased sarcomere lengths observed at 2 weeks and not 20 weeks.

Desmin, is an intermediate filament protein and has an essential role in maintaining the spatial relationship between the contractile apparatus and other structural components of the cardiomyocytes such as the sarcolemma, mitochondria, and nucleus (28–30). This protein mechanically links the Z-disc to the costameres, and thus, desmin is concentrated at the Z-line and the intercalated disc in healthy cardiomyocytes. After the onset of MR, irregularity and loss of desmin staining was evident across the length of the myocyte. Focal concentration of desmin at the intercalated disc was pronounced after 2 and 10 weeks of MR compared with the control group, indicating redistribution of desmin with acute and chronic exposure to MR. Previous work has shown that the mechanism of cardiomyocyte elongation occurs through sarcomere addition at the intercalated disc region (15). When there is loss of desmin at the intercalated disc, this has been associated with arrhythmias and sudden cardiac death (31). Thus, rearrangement and increased concentration of desmin at the intercalated disc region may help to stabilize this remodeling region of the elongating cardiomyocyte in the setting of MR.

The cytoskeletal network of proteins structurally impacts the mitochondria, regulating its structure, spatial organization within the cells, and its function (32, 33). In this study, we observed that volume overload from MR caused a loss of the linear registry of the mitochondria, clustering around the sarcomeres and nucleus, and irregularities in the cristae, which are the primary location for oxidative phosphorylation and ATP production. Similar findings have been reported wherein cytoskeletal breakdown parallels mitochondrial dysfunction in the ACF model, despite preserved ejection fraction (34). These ultrastructural alterations to the mitochondria may contribute to the development of oxidative stress, which is known to occur in response to volume overload (34–37). In a recent study, we reported that MR leads to an increase in pressure-volume loop area, indicative of increased myocardial oxygen consumption, and an activation of oxidative stress related genes (9), which could be a result of the increased myocardial energetic demand and structural adaptations of the mitochondria. Despite preserved ejection fraction in the setting of MR, these adverse mitochondrial and cytoskeletal changes could be a precursor to mitochondrial dysfunction and eventual LV dysfunction. Lastly, the increased presence of autophagic vacuoles at both early and later time-points after the onset of MR could indicate cardiomyocyte autophagy from increased mechanical stretch, hypertrophy, or the development of heart failure (38, 39). Glycogen accumulation observed after 2 weeks of MR is indicative of a stress-induced metabolic state of the cardiomyocytes, that has manifested at the ultrastructural level. Unlike the normal heart where ATP production is primarily dependent on fatty acids, the remodeling, hypertophic heart can revert back to a fetal-like metabolic state in which ATP production is reliant on glucose for which the cardiomyocyte can quickly access glycogen for the production of ATP when metabolic demand is high (40).

The findings of this study should be considered with some limitations of the experimental work. In this study, we retrospectively studied the cardiomyocyte ultrastructure and cytoskeletal proteins in the animals, and thus were limited to the available sample size. Rationing these hearts for TEM and immunohistochemistry studies, due to differences in their tissue processing, further limited our sample size for each assay. Therefore, the sample size does not enable a paired analysis approach to relate ultrastructure to cardiac function, which we intend to pursue in future work with a larger sample size. We report alterations of the I-band feature of the sarcomere after the onset of MR, however, these cannot definitively prove whether altered levels of titin play a role in MR-induced cardiac remodeling in this model of severe MR. Investigation of titin and other cytoskeletal proteins would contribute to the understanding of the cardiomyocyte structure-function relationship as the heart remodels in this specific lesion. Lastly, whether or not the ultrastructural alterations in the cardiomyocytes are reversible even after the correction of MR is still not known. Future work would be necessary to understand the impact of MR correction and the timing of MR correction on the underlying cellular level and function of the cardiomyocytes. Future studies should also investigate whether emerging therapeutics targeting the cytoskeleton or mitochondria could potentially halt adverse remodeling.

## Data Availability Statement

The original contributions presented in the study are included in the article/supplementary material, further inquiries can be directed to the corresponding author.

## Ethics Statement

The animal study was reviewed and approved by Institutional Animal Care and Use Committee at Emory University.

## Author Contributions

DC and MP defined the hypothesis and designed the experiments. DC conducted the experiments, analyzed the data, and prepared the manuscript. MP reviewed the data, edited the manuscript, acquired the funding and resources to complete the work, and approved the final version for submission. AS interpreted the electron microscopy images and provided written expert reports in a blinded manner. All authors contributed to the article and approved the submitted version.

## Funding

This work was funded by grants HL133667, HL135145, and HL140325 from the National Heart, Lung and Blood Institute, a predoctoral fellowship Grant 19PRE34380625 from the American Heart Association and infrastructure support from the Carlyle Fraser Heart Center at Emory University Hospital Midtown.

## Conflict of Interest

MP is a consultant to Heart Repair Technologies Inc and Boston Scientific, and receives consulting fees. MP also discloses a significant stock ownership in Nyra Medical Inc. The remaining authors declare that the research was conducted in the absence of any commercial or financial relationships that could be construed as a potential conflict of interest.

## Publisher's Note

All claims expressed in this article are solely those of the authors and do not necessarily represent those of their affiliated organizations, or those of the publisher, the editors and the reviewers. Any product that may be evaluated in this article, or claim that may be made by its manufacturer, is not guaranteed or endorsed by the publisher.
